# Plasma-Derived Inflammatory Proteins Predict Oral Squamous Cell Carcinoma

**DOI:** 10.3389/fonc.2018.00585

**Published:** 2018-12-04

**Authors:** Kelly Yi Ping Liu, Xian Jun David Lu, Yuqi Sarah Zhu, Nhu Le, Hugh Kim, Catherine F. Poh

**Affiliations:** ^1^Department of Oral Biological and Medical Sciences, Faculty of Dentistry, University of British Columbia, Vancouver, BC, Canada; ^2^Department of Integrative Oncology, British Columbia Cancer Research Centre, Vancouver, BC, Canada; ^3^Department of Cancer Control Research, British Columbia Cancer Research Centre, Vancouver, BC, Canada; ^4^Centre for Blood Research, University of British Columbia, Vancouver, BC, Canada; ^5^Department of Biochemistry and Molecular Biology, University of British Columbia, Vancouver, BC, Canada; ^6^Department of Pathology and Laboratory Medicine, University of British Columbia, Vancouver, BC, Canada

**Keywords:** oral carcinogenesis, biomarker, plasma, inflammation, protein expression, clinical outcomes

## Abstract

Oral squamous cell carcinoma (OSCC) is a major concern with high morbidity and mortality worldwide, even with the current knowledge and the advancement in treatment. OSCCs diagnosed at late-stage often require wide-excision with or without neck dissection, radiotherapy, or chemotherapy. When deemed successful, treatment often results in diminished quality of life, impaired function, and disfigurement. Strategies for early detection are urgently needed for patients afflicted with this disease. Inflammatory protein plasma biomarkers have shown to be potential tests for early detection and disease monitoring in several cancers. There has been no study on inflammation-related plasma biomarkers in OSCC. The objectives of the study were to use a multiplex approach to screen plasma-derived biomarkers and to examine the association of measurable proteins with OSCC. A total of 260 plasma samples (210 OSCC and 50 normal controls) were collected to measure for concentration of inflammatory related biomarkers using electrochemiluminescence multiplex assay. After screening of 82 potential biomarkers of the first 160 OSCC, 16 cytokines, chemokines, and growth factors were identified and verified in the second set of samples containing 50 OSCC and 50 normal. After adjustment of age and batch effects, the adjusted differential expression analysis showed that the OSCCs were markedly lower in 14 biomarkers and significantly higher level of interleukin 1 receptor antagonist (IL1Ra). By performing unsupervised clustering analysis, we observed distinctive groups of normal and two subgroups of OSCC. Linear regression of IL2, IL1Ra, and macrophage inhibitory factor (MIF) showed high accuracy in classifying OSCC with sensitivity of 0.96 and specificity of 0.92. In conclusion, this is the first paper to identify potential inflammatory plasma protein biomarkers of patients with OSCC. With further validation, the set of biomarkers can potentially be used to assist in early detection of OSCC when the disease is localized and in more treatable stage.

## Introduction

Oral squamous cell carcinoma (OSCC), the most common form of oral cancer, remains a global health issue accounting for 274,000 new cases and 145,000 deaths each year (1, 2). Despite advancement in treatment, the improvement of 5-year survival rates (30–60%) is diminutive mainly due to the aggressive nature of this disease and its high recurrence rates in lymph nodes and distant organs (3, 4). Early detection of cancerous lesions at more localized and treatable stages can potentially improve this decimal outcome. However, OSCCs are often caught at late stage which largely relies on regular screenings with invasive diagnostic biopsies. In addition, post-treatment complications include scarring and trauma which often cause tissue alterations and can preclude identification of early recurrence. Moreover, repeated biopsies for post-treatment monitoring is impractical and can further traumatize the yet-to-heal mucosal surface. Therefore, there is a need for a non-invasive tool for early detection of OSCC to improve clinical treatment and patient's quality of life.

The advancement of genomic technologies has made it possible for early detection of key biological events that contribute to tumorigenesis of OSCC. It is largely accepted that OSCC, like other cancers, is a genetic disease characterized with loss of heterozygosity (5, 6), deregulation of cell cycle or proliferation proteins expression (7–10), and dysregulation of microRNA expression (11–14). Harnessing the immune response directed against tumors is another promising event given the well-established evidence of immune-related molecules reacting toward tumor antigens in a variety of cancer types. Given such, identification of biomarkers that are specific to the OSCC environment would provide an effective strategy for cancer screening.

The circulatory system has been known to constitute of components that reflect diverse physiological and pathological states. Therefore, the sampling of blood, as opposed to tissue biopsies, is an attractive avenue for developing a relatively less-invasive screening test, especially with the advent of proteomic technologies such as mass spectrometry or microarray-based assays. Previous studies comparing serum or plasma levels between healthy controls, premalignant, and OSCC have revealed significant differences in several proteins such as angiogenic factor (15–17), cytokines, chemokines, and growth factors (18–21). The objectives of this study were to use a multiplex approach to screen plasma-derived biomarkers and to examine the association of measurable proteins with OSCC.

## Materials and Methods

### Study Population

The OSCC patients were identified from a pan-Canadian surgical trial (NCT01039298) (22). Among the 443 patients enrolled between 2010 and 2016, we identified 210 OSCCs from the oral anatomical sites (ICD-10 site codes of C02.0—C06.9) with at least 3 years of post-surgery follow-up. The blood samples were collected at time of surgery, processed within 4 h of collection, and had not gone through any freeze-thaw cycle. Patient baseline demographic data included age, sex, ethnicity, smoking history, exposure to second-hand smoke, and alcohol consumption. Clinical-pathological data included lesion anatomical site, clinical assessment of tumor size and neck lymphadenopathy, tumor grade, depth of invasion. Outcome data included overall survival, disease-specific survival, and development of nodal disease during post-surgery follow-up.

**Figure 1:**
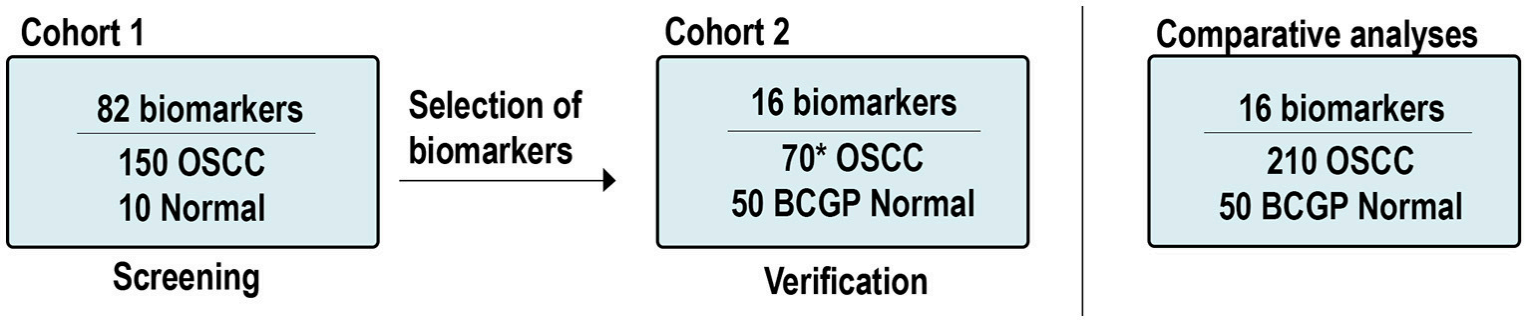
Study scheme and samples. *Includes: 60 new OSCCs and 10 OSCCs from Cohort 1. OSCC, oral squamous cell carcinoma; BCGP, British Columbia Generations Project.

In addition to the OSCC samples, there were 60 normal samples used. Ten samples were from existing normal blood samples collected for other studies and served as a baseline normal for Cohort 1; 50 plasma samples from participants of the British Columbia Generations Project (BCGP) were requested based on one-to-one matching criteria for age (±5 years old), sex, and smoking history. These participants, recruited between 2010 and 2016, had no known cancer history at the time of blood collection up to the last data update in November 2017. Pre-analytical conditions were the same as those of the OSCC samples, including processing whole-blood samples within 4 h of collection and samples had not gone through any freeze-thaw cycle. These BCGP samples served as baseline normal in comparative analyses. Supplementary Tables 1, 2 summarize the study population by Cohort. The study schema is illustrated in Figure 1.

This study was carried out in accordance with the recommendations of University of British Columbia Clinical Research Ethics and the BC Cancer Research Ethics General Guidance Notes (GNs), BC Cancer Agency Research Ethics Board. This study utilized the clinical information and samples collected from existing studies which were approved by the BC Cancer Agency Research Ethics Board (REB#H09-03090 and REB#H08-01354, respectively). The present study was approved under REB#17-02031.

### Sample Preparation

Whole blood samples were collected in EDTA vacutainer tubes (Becton Dickinson, Franklin Lakes, NJ, USA), either at time of surgery for OSCC or at the time of enrollment to BCGP, stored at 4°C, and processed within 4 h of collection. The OSCC whole blood samples were centrifuged at 1,500 × g for 15 min at room temperature to separate blood plasma which were then stored at −80°C until usage. The BCGP whole blood samples were processed for plasma separation in accordance to BCGP's standard operating protocol, with centrifugation at 1,300 × g for 10 min at 4°C (23).

### Electrochemiluminescence Multiplex Assays

Plasma protein expression was measured based on multiplex electrochemiluminescence (ECL) detection assays using commercially available kits from Meso Scale Diagnostics (MSD) (Rockville, MD, USA). We first screened potential OSCC biomarkers among the 82 biomarkers across Cohort 1 (150 OSCC and 10 normal; Figure 1). These 82 biomarkers were included in the V-PLEX Angiogenesis Panel 1 Human Kit (*n* = 3, K15190D), V-PLEX Vascular Injury Panel 2 Human Kit (*n* = 5, K15198D), U-PLEX TGF-β Combo Human (*n* = 3, K15241K), and U-PLEX Biomarker Group 1 Human (*n* = 71, K15081K). The screening results from Cohort 1 are summarized in Supplementary Table 3.

Based on the differential expression analysis from Cohort 1 and the current literatures (24–26), we identified 16 candidate biomarkers and verified them by performing ECL assays (V-PLEX Angiogenesis Panel 1 Human Kit, *n* = 3; V-PLEX Vascular Injury Panel 2 Human Kit, *n* = 3; U-PLEX Biomarker Group 1 Human, *n* = 10) on Cohort 2 (60 OSCC and 50 BCGP normal; Figure 1). The verification results of 16 biomarkers in Cohort 2 are summarized in Supplementary Table 3. To assess the assay's reproducibility, we also randomly selected 10 OSCC samples from Cohort 1 and repeated the measurement for the 16 candidate biomarkers as part of Cohort 2.

All ECL assays were conducted as per the manufacturer's protocols. Briefly, supplied 96-well plates were washed and coated (for U-PLEX kits) with monoclonal antibodies followed by addition of serially diluted calibrator standard in duplicates and plasma samples (20 to 50 μL with dilution factor as per assay protocol), incubation with shaking (1–2 h, room temperature), washing (three times each well), addition of 20–50 μL SULFO-TAG conjugated secondary monoclonal antibodies, and final incubation with shaking (1–2 h, room temperature). MSD Read Buffer was added to each well right before loading the plates for signal detection on the QuickPlex SQ 120 (Meso Scale Diagnostics, LLC, Rockville, MD, USA). Pre-analytical data processing was performed on MSD Discovery Workbench software version 4.0.12 to calculate the concentration of each biomarker in each sample based on the standard curves generated from calibration standards using the four-parameter logistic fit.

### Statistical Analysis

All data analysis was performed using R version 3.4.4. For comparative analyses, we considered the BCGP samples as the “normal” group as opposed to the “diseased” OSCC group. Patient demographics and clinical-pathological characteristics were compared by using Student's *t-*test for continuous variables or Fisher's exact test for categorical variables. All statistical tests at *p* < 0.05 were considered significant.

To screen for potential OSCC biomarkers, we performed unpaired two-group Wilcoxon Mann-Whitney test to compare the concentration level between OSCC and normal in Cohort 1. Those with *p* < 0.05 after correction for multiple testing with Benjamin-Hochberg (BH) procedure were considered significant as candidate biomarkers for verification in Cohort 2. Finally, differential expression analysis on the 16 candidate biomarkers was performed on the 210 OSCC against 50 BCGP normal.

To examine the association between biomarker concentrations and OSCC, we first used logistic regression analyses to assess the potential impact of patient demographic variables, selecting those with *p* < 0.05 as potential confounding factors. Linear regression analyses for differential expression between OSCC and normal were then performed for each biomarker, adjusting for confounding variables and batch-effect that may hinder with clustering analysis.

To investigate presence of subgroups of samples, we used hierarchical clustering (pheatmap v1.0.10) (27) with input as the concentration (pg/mL) data matrix for the candidate biomarkers across the 210 OSCC and 50 normal samples to identify subgroups within the study population. We used Ward.D2 for the clustering method with Pearson correlation and Euclidean as the distance measures for clustering the columns and rows respectively. Further, the relationship among candidate biomarkers in OSCC or in normal samples is presented by network visualization (qgraph v1.5) (28) with input of Pearson correlation coefficient matrix of log10 transformed concentration. The output is a network composed of circles of nodes, which each represents a candidate biomarker, connected by lines that represent strength of significant correlation with *p* < 0.05, i.e., the greater the distance between two biomarkers, the lower the correlation and absence of connecting lines denotes zero correlation. The placement of the nodes represents how biomarkers cluster.

To explore the potential of these biomarkers in detection of OSCC, we first performed LASSO penalized regression analysis (glmnet v2.0-16) (29) to identify biomarkers and baseline covariates that best classify OSCC with highest accuracy. The regression model input was log10 transformed biomarker concentration. We randomly partitioned the entire study population (*n* = 260) into training set for model development and test set for evaluation of the fitted model. The biomarkers with highest discriminative performance were tested for classification performance by computing their sensitive and specificity in classifying normal and OSCC in the test set. A receiver operating characteristic (ROC) curve was then generated (pROC 1.12.1) (30) with area under the curve (AUC) estimation of predictability for OSCC.

## Results

### Study Population

Patient demographics are summarized in Table 1 (OSCC and BCGP normal) and tumor characteristics are summarized in Supplementary Table 2. OSCC patients were mainly middle-aged, ever-smoked, and White; compared to BCGP participants, the OSCC patients were older (*p* < 0.001) (Table 1). Majority of OSCC lesions were on the tongue (66.7%) and early-staged (72.3%). In addition, 33.3% of the patients had loco-regional recurrence, 18.1% died of disease, and 10.9% died of other cause (Supplementary Table 2). We performed multivariate logistic regression analysis to assess the potential association of demographic variables with OSCC (Table 1). Age and ethnicity were significantly associated with OSCC.

**Table 1:** Demographics of study population.

						**Multivariate logistic regression**	
**Variables**	**Total (*n* = 260)**	**BCGP (*n* = 50)**	**OSCC (*n* = 210)**	**Early-stage OSCC^a^ (*n* = 152)**	**Late-stage OSCC^a^ (*n* = 58)**	**OR (95% CI)**	***p^*c*^***
Age, mean ±*SD*	62.4 ± 13.5	56.8 ± 8.27	63.8 ± 14.2	63.9 ± 14.4	63.3 ± 13.9	1.0 (1.0–1.0)	< 0.001
Age, median (1–3 Qtile)	62 (53.8–71.4)	60 (49.0–62.0)	63.7 (54.5–75.4)	63.5 (54.5–75.5)	64.2 (55.3–73.4)	
**AGE GROUP**						
< 50	56 (21.5)	14 (28.0)	42 (20.0)	31 (20.4)	11 (19.0)	1
50–62	77 (29.6)	24 (48.0)	53 (25.2)	37 (24.3)	16 (27.6)	0.74 (0.3–2.1)	0.56
>62	127 (48.8)	12 (24.0)	115 (54.8)	84 (55.3)	31 (53.4)	3.1 (1.0–9.3)	0.04
**SEX**						
Male	139 (53.5)	29 (58.0)	110 (52.4)	75 (49.3)	35 (60.3)	1
Female	121 (46.5)	21 (42.0)	100 (47.6)	77 (50.7)	23 (39.7)	1.3	0.58
**ETHNICITY**						
White	194 (74.6)	42 (84.0)	152 (72.4)	107 (70.4)	45 (77.6)	1
Other^b^	66 (25.4)	8 (16.0)	58 (27.6)	45 (29.6)	13 (22.4)	3.6 (1.3–10.7)	0.01
**SMOKING HISTORY**						
Never	122 (46.9)	25 (50.0)	97 (46.2)	71 (46.7)	26 (44.8)	1
Current	63 (24.2)	14 (28.0)	49 (23.3)	32 (21.1)	17 (29.3)	0.9 (0.4–2.6)	0.90
Former	71 (27.3)	11 (22.0)	60 (28.6)	45 (29.6)	15 (25.9)	1.3 (0.4–3.6)	0.66
Unknown	4 (1.5)		4 (1.9)	4 (3.0)		

a *OSCC patients were categorized based on the AJCC 8th Edition Cancer Staging System for head and neck cancers (31). Early-stage OSCC consists of T1 or T2 with depth of invasion (DOI) < 10 mm; late-stage OSCC consists of T3 or any tumor >10 mm DOI, or T4, or lymph node positive*.

b *Other ethnicity includes Aboriginal (n = 2, early-stage OSCC) and Asian (n = 8, BCGP; n = 43, early-stage OSCC; n = 13, late-stage OSCC)*.

c Statistical analysis was performed excluding unknown data (n = 4, Smoking History)

*BCGP, British Columbia Generations Project; OSCC, oral squamous cell carcinoma; OR, odds ratio; CI, confidence interval*.

### Differential Expression of Plasma Biomarker Level Between OSCC and Normal

We first screened for potential candidate biomarkers from Cohort 1 (150 OSCC and 10 normal) by comparing the concentration of 82 biomarkers using unpaired two-group Wilcoxon Mann-Whitney Test (Supplementary Table 3). The results showed 16 biomarkers with *p* < 0.05 after correction for multiple testing. Among these, we selected bFGF, CRP, I309, ICAM1, IL10, IL1a, IL1Ra, IL2, IL6, MCP3, MCSF, MIF, MIP1a, SAA, Tie2, and VEGFD as candidate biomarkers for further verification in Cohort 2 (Supplementary Table 3).

**Figure 2:**
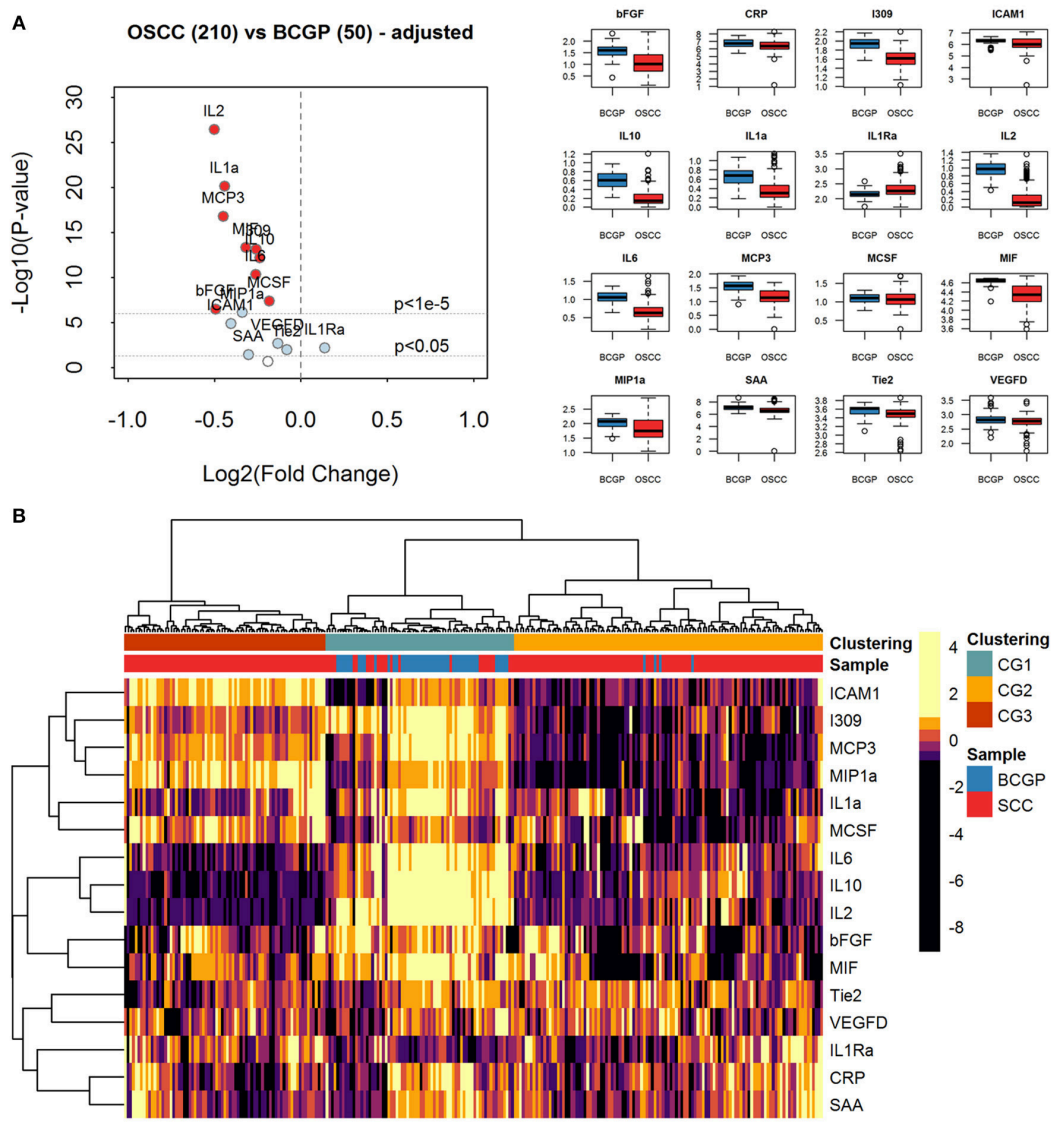
Expression analysis of plasma biomarkers. **(A)** Volcano plot of log2 transformed fold change vs. log10 transformed *p*-values showing differential expression for each biomarker across comparative sample set (*n* = 260) calculated by linear regression tests with adjustment of age, ethnicity, and batch-effect. Significantly differentially expressed biomarkers are labeled with light blue solid dots for *p* < 0.05 and red solid dots for *p* < 1e-5. The distribution of concentration (log10(pg/ml+1)) is summarized with boxplots displayed in alphabetical order of biomarker. **(B)** Heatmap of unsupervised hierarchical clustering of biomarkers across the comparative sample set (*n* = 260). Column labels represent the disease group of samples: Blue: BCGP; Red: OSCC; and 3-group (*k* = 3) clustering by Pearson correlation and Euclidean distance measurement: Green, Clustering Group (CG1) (*n* = 70), Orange: Clustering Group (CG2) (*n* = 115); Brown, Clustering Group (CG3) (*n* = 75).

To assess the differential expression between 210 OSCC and 50 BCGP normal for the 16 candidates, we performed linear regression analysis with adjustment for age, ethnicity, and batch as confounding variables (Table 2). This revealed 15 candidate biomarkers that were significantly differentially expressed (*p* < 0.05) with 14 significantly lower and IL1Ra significantly higher in OSCC samples, comparing to BCGP normal (Table 2 and Figure 2A). Given the objective was to identify biomarkers for early detection of OSCC, we also performed differential expression analysis between normal (*n* = 50) and early-stage OSCC (T1/T2 and DOI ≤ 10 mm; *n* = 152) (31), and between early-stage and late-stage OSCC (T3/T4 and/or DOI>10 mm, *n* = 58) (Table 1). We observed similar results in differences between normal and early-stage, but there was significantly higher concentration of CRP and SAA in late-stage OSCC (Supplementary Tables 5, 6).

**Table 2:** Differential expression analysis between OSCC and BCGP samples.

**Biomarker^a^**	**Total (*****n*** = **260)**		**BCGP (*****n*** = **50)**		**OSCC (*****n*** = **210)**			
	**Mean ±*SD***	**Median (1Q−3 Qtile)**	**Mean ±*SD***	**Median (1Q−3 Qtile)**	**Mean ±*SD***	**Median (1Q−3 Qtile)**	**Fold change^b^**	***p^*b*^***
bFGF	1.2 ± 0.5	1.2 (0.8–1.6)	1.7 ± 1.6	1.6 (1.4–1.7)	1.1 ± 0.5	1.0 (0.7–1.4)	0.61	< 0.0001
CRP	6.5 ± 0.7	6.4 (6.0–6.9)	7.1 ± 7.2	6.7 (6.4–7.2)	6.4 ± 0.7	6.4 (6.0–6.9)	0.82	0.2
I309	1.7 ± 0.2	1.7 (1.5–1.8)	1.9 ± 1.4	1.9 (1.8–2.0)	1.6 ± 0.2	1.6 (1.5–1.7)	0.77	< 0.0001
ICAM1	6.1 ± 4.9	6.1 (5.8–6.5)	6.3 ± 6.1	6.3 (6.2–6.4)	6.1 ± 0.5	6.0 (5.8–6.5)	0.67	< 0.0001
IL10	0.3 ± 0.2	0.2 (0.1–0.4)	0.5 ± 0.3	0.5 (0.3–0.7)	0.2 ± 0.2	0.1 (0.09–0.3)	0.79	< 0.0001
IL1a	0.4 ± 0.3	0.4 (0.2–0.7)	0.6 ± 0.3	0.6 (0.4–0.7)	0.3 ± 0.2	0.3 (0.2–0.5)	0.64	< 0.0001
IL1Ra	2.3 ± 0.3	2.2 (2.1–2.4)	2.2 ± 1.7	2.5 (2.1–2.2)	2.3 ± 0.3	2.3 (2.1–2.4)	1.15	0.006
IL2	0.4 ± 0.4	0.2 (0.06–0.7)	1 ± 0.6	0.9 (0.8–1.1)	0.2 ± 0.3	0.1 (0.03–0.3)	0.61	< 0.0001
IL6	0.7 ± 0.3	0.7 (0.6–0.9)	1 ± 0.7	1.0 (0.9–1.1)	0.7 ± 0.2	0.6 (0.5–0.8)	0.77	< 0.0001
MCP3	1.2 ± 0.3	1.3 (1.0–1.5)	1.6 ± 1.3	1.6 (1.4–1.7)	1.2 ± 0.3	1.1 (1.0–1.4)	0.64	< 0.0001
MCSF	1.1 ± 0.2	1.1 (0.9–1.2)	1.1 ± 0.6	1.1 (1.0–1.2)	1.1 ± 0.2	1.1 (0.9–1.2)	0.83	< 0.0001
MIF	4.4 ± 0.2	4.4 (4.2–4.6)	4.6 ± 3.8	4.6 (4.6–4.7)	4.3 ± 0.2	4.3 (4.2–4.5)	0.76	< 0.0001
MIP1a	1.9 ± 0.4	1.8 (1.6–2.1)	2.1 ± 1.7	2.1 (1.9–2.2)	1.8 ± 0.4	1.7 (1.5–2.1)	0.71	< 0.0001
SAA	6.7 ± 0.7	6.7 (6.4–7.1)	7.4 ± 7.8	7.1 (6.8–7.3)	6.6 ± 0.8	6.6 (6.3–7.0)	0.74	0.03
Tie2	3.5 ± 0.2	3.5 (3.4–3.6)	3.6 ± 3	3.6 (3.5–3.6)	3.5 ± 0.2	3.5 (3.4–3.6)	0.92	0.009
VEGFD	2.8 ± 0.2	2.8 (2.7–29)	2.9 ± 2.8	2.8 (2.7–2.9)	2.8 ± 0.2	2.8 (2.7–2s.9)	0.87	0.002

a *Biomarker mean and median was calculated from log10 (pg/ml+1) transformed measurements*.

b *Differential expression analysis was adjusted for age, ethnicity, and batch-effect*.

### Unsupervised Clustering of Biomarker Expression Reveals Subgroups of Samples

To investigate the extent of heterogeneity of biomarker across the 260 samples, we performed unsupervised hierarchical clustering which revealed 3 main clustered groups (CGs) of samples (Figure 2B). The CG1 comprised mainly normal (65.7%) while most of the OSCC were clustered into CG2 and CG3, with CG2 showing distinctively lower levels of ICAM1, I309, MCP3, MIP1a, and IL1a. Comparing between CG2 and CG3, similar baseline demographics and clinical-pathological characteristics were observed (Supplementary Table 4), suggesting that there are other clinical or biological factors associated with the clustering. Interestingly, compared to CG3, there were more CG2 OSCC with greater tumor size of T3/T4 (9.9 vs. 5.3%) and lymph node positive at time of surgery (21.7 vs. 13%), and that significantly more late-stage OSCC were in CG2 (*p* = 0.005). This suggests that the plasma level of these biomarkers may infer the staging of tumor at time of initial diagnosis.

**Figure 3:**
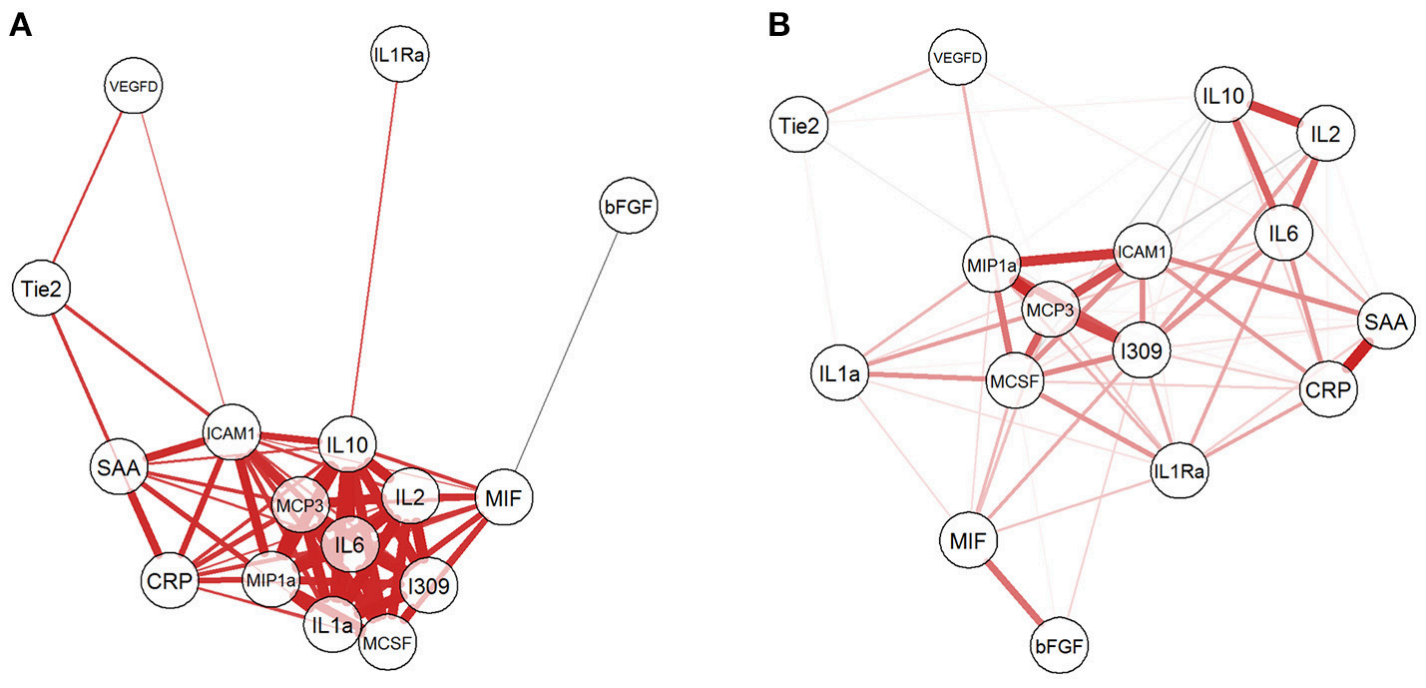
Network visualization of correlation between biomarkers. For both BCGP **(A)** and OSCC **(B)** networks, each biomarker is a circle with lines representing significant correlation (Student's *t*-test, alpha = 0.05) between biomarkers, with red and black for positive and negative correlation, respectively. The line thickness represents the strength of the correlation, the thicker the lines, the stronger the correlation and vice versa.

Given that the biomarkers were also clustered into three groups by hierarchical clustering, we investigated the relationship among them by computing Pearson correlation coefficients with correlation network visualization (Figure 3). The network of BCGP normal (Figure 3A) consisted of strong and tight correlations (thick red lines) for most of the biomarkers, except MIH and bFGF showing negative correlation (thin faded black line). In contrast, OSCC (Figure 3B) showed 2 tight clusters (MCP3, I309, ICAM1, MIP1a, and MCSF; IL2, IL6, and IL10) with less number of biomarkers, a separate but close relationship between MIF and bFGF, and negative correlations of ICAM to IL2/IL10, I309 to Tie2, and MCSF to IL2/IL10, and MIP1a to IL10 (faded black lines). This suggests that there are subgroups of OSCC which may differ in biological processes introducing the noise to the relationship between these biomarkers. In addition, there was an inverse relationship for several markers where the correlation is positive in BCGP normal but negative in OSCC. This may also reflect the consequences of response mechanism of certain cytokines in presence of a tumor.

### Discriminative Performance of Plasma Biomarkers

**Figure 4:**
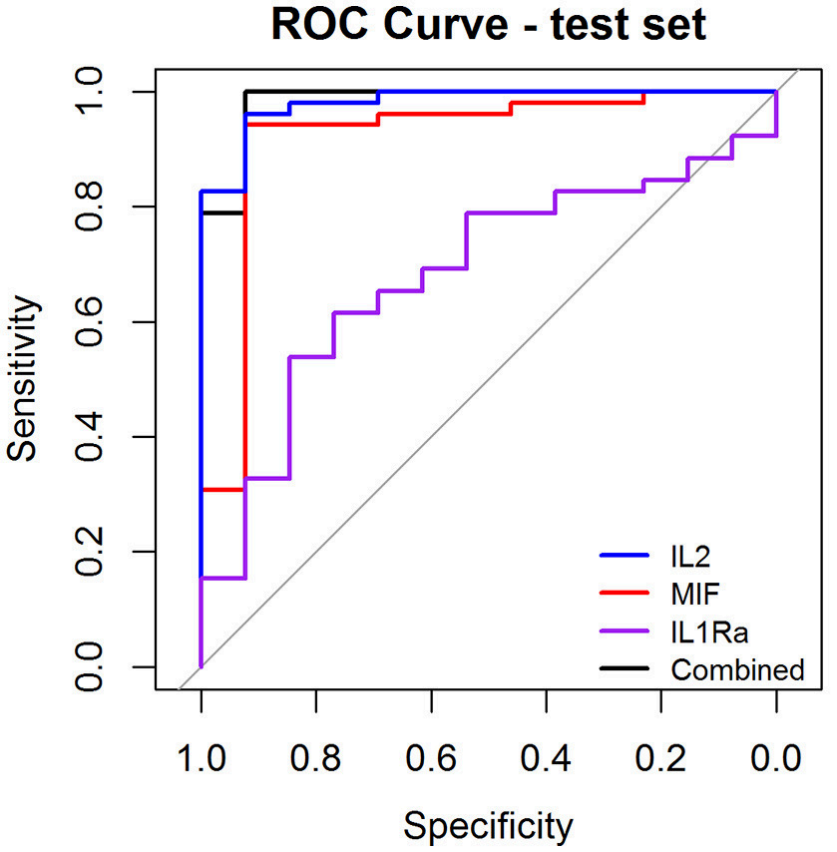
Receiver Operator Characteristic (ROC) curves illustrating classification performance of modeled biomarkers on OSCC vs. BCGP normal among the test set. The curves illustrate performance range for each of the biomarkers (IL2, blue line; MIF, red line; IL1Ra, purple line) The combination of the 3 biomakers (black line) achieved AUC of 0.95 (95% CI, 0.87–0.99).

As a preliminary step to investigate the diagnostic performance of these circulating biomarkers, we randomly partitioned the 260 samples into training (*n* = 195, 158 OSCC and 37 normal) and test (*n* = 65, 52 OSCC and 13 normal) sets. LASSO penalized regression was performed on the training set which selected MIF, IL2, and IL1Ra as variables that best classified OSCC and normal. These selected variables were then applied to the test set. Although the test set was small, the model achieved high performance with AUC of 0.96, sensitivity of 0.96, specificity of 0.92, PPV of 0.98 and NPV of 0.86 (Table 3 and Figure 4).

**Table 3:** Discriminative performance of biomarkers.

**Biomarker(s)**	**AUC (95% CI)**	**Se**.	**Sp**.	**PPV**	**NPV**
IL2	0.9077 (0.8098–0.9654)	0.9038	0.9231	0.9792	0.7059
IL2+MIF	0.9538 (0.871–0.9904)	0.9615	0.9231	0.9804	0.8571
IL2+MIF+IL1Ra	0.9538 (0.871–0.9904)	0.9615	0.9231	0.9804	0.8571

*AUC, area under the ROC curve; CI, confidence interval; Se., sensitivity; Sp., Specificifiy; PPV, positive predictive value; NPV, negative predictive value*.

## Discussion

OSCC, with poor survival and significant impact on quality of life, has been an under-studied disease. As an immune inhibitory disease, it is known to be associated with increased expression of cytokines and chemokines at the tumor microenvironment with mounting evidence associating these inflammatory changes with stages of diseases and recurrence (32, 33). Therefore, circulating biomarkers may also reflect the pathological disease states and blood samples which constitute detectable proteins can potentially be used to screen for OSCC. By comparing to normal samples, this is the first study to identify a set of potential plasma inflammatory protein biomarkers to distinguish OSCC from normal.

Sampling of blood is a more convenient and a relatively less invasive means of sample collection compared to invasive tissue biopsies. In addition, examining blood protein components provides an indication of a systemic analysis of the changes in the presence of cancer. Comprehensive proteomics approach is suitable for biomarker discovery; however, it can be costly in conducting the assay and executing proteomic analysis while integrating sources of variables contributing to aberrantly expressed proteins. The MSD, an ECL-based platform, is commercially available and can be customized to screen or measure a set of targeted protein expressions, and has shown promising robust results for screening of diseases (34, 35). Moreover, compared to the enzyme-linked immunosorbent assay (ELISA), ECL has been found to have higher sensitivity with capability of multiplex up to ten biomarkers per sample. As the first step of biomarker screening, we used MSD as our approach to screen for potential targets and verify them in an independent set of test samples. In our study, we have assessed the potential confounding impact on age. Comparing to the normal controls after adjustment for age and batch effects, all differentially expressed biomarkers remained significant.

Our study samples were subjected to only 1 to 2 freeze-thaw cycles. The process of freeze-thawing or storage time has been investigated for its effect on detectable concentration of blood proteins. Studies have found that freeze-thaw, up to a maximum of five cycles, and long-term storage of samples at −80°C had minimum changes to the concentrations of blood plasma proteins (36–38). A recent study investigated the effects of freeze-thaw by comparing concentrations of inflammatory proteins, of which 9 overlap with our panel (39). No significant differences were found between never-frozen and at least one freeze-thaw cycle. For a blood-based diagnostic test in clinical settings, immediate processing of fresh samples is convenient. However, to be cost-effective in clinical settings, MSD assay should analyze 80 collected samples. Thus, subjecting samples to at least one freeze-thaw cycle may be unavoidable. Nevertheless, if the plasma biomarkers in this study are validated as OSCC specific, more economical clinical platforms, such as ELISA, can be designed to test on fresh-blood samples in clinical settings.

The most intriguing observation from this study is the significant low level of these biomarkers in OSCC, compared to normal samples. Although this was unexpected, OSCC is known to be an immune inhibiting disease; therefore, the observed low-level expression may reflect this. Another possible explanation is that tumor-tissue associated inflammatory-related proteins are present at higher concentration at the tumor tissues than in the blood. Further investigation of the expression of these targets at the tumor tissue, unaffected tissues, and correlated to the circulating levels may shed light of its underlying mechanism. However, given the multifaceted nature of these biomarkers, in which expression is affected by a variety of signaling pathways, the underlying biological explanation of the observed expression requires further experiments which are beyond the scope of this study. Nevertheless, with the observed blood level we were able to estimate the performance in classifying OSCC which achieved high sensitivity and specificity.

The present study found strong association between OSCC and decreased level of proteins involved in immune response, including IL2, MIF, and IL1Ra. IL2 is one of the key cytokines with regulatory role in T-cell expansion and activation through main signaling pathways (STAT, PI3K-AKT, and MAPK) that mediates the survival, proliferation, differentiation, activation, and cytokine production in different types of immune cells (40, 41). IL2 is predominantly produced by antigen-stimulated T cells, NK cells, and activated dendritic cells. The absence of IL2, thus, infers the characteristic of immune deficient head and neck cancer, including OSCC (42). We observed almost undetectable trace of circulating IL2 in OSCC.

MIF is a pro-inflammatory cytokine constitutively expressed and readily to be secreted by activated immune cells promoting cell proliferation and angiogenic activities, facilitating detection of antigens, and production of other inflammatory cytokines (43). In regard to carcinogenesis, high expression of MIF has been found to inhibit regulatory effects of p53 mediated apoptosis in tumor-cell lines, and cytotoxic CD8+ T cells (44, 45). In addition, MIF was also demonstrated to activate T cell through production of pro-inflammatory molecules, including IL2 and IL6 (44). The low expression of MIF may explain the observed low level of IL2.

IL1Ra is structural variant of IL1 ligand with anti-inflammatory effects by competitively binding to IL1 receptors. Therefore, the elevated level of IL1Ra in circulation may indicate the presence of inflammatory effects of IL1 in tumor tissues which trigger the IL1Ra to counterbalance the signaling pathways activated by IL1. This suggests expression of IL1Ra plays a role in demoting the progression of tumor (46). Several studies have demonstrated the expression of IL1Ra to be positively correlated with progression and lymph node metastasis (47–49), inhibit IL-1 mediated prostate cancer regression (50), and increased growth rate of glioblastoma cells (51). In our study, the expression of IL1Ra is markedly higher in patients with OSCC compared to normal controls. In addition, we observed significantly higher level of IL1Ra in OSCCs that developed lymph node disease (fold change 1.1, *p* = 0.03). These results may be suggesting that an increase in IL1Ra was to reduce the tumor-mediated production of IL1 (52) and could propose value in assessing disease severity.

To explore the correlation among the candidate biomarkers using network visualization, we have observed interesting reverse relationships between biomarkers between OSCC and normal BCGP samples. For example, bFGF was negatively correlated with MIF among normal BCGP samples but showed significant positive correlation between MIF and several other biomarkers. This reflects previous reports demonstrating the production of MIF in presence of growth factors and inducing tumor growth (53, 54). We also observed significant negative correlation between ICAM1 and IL10 among the OSCC samples suggesting the inhibitory role of IL10 on ICAM1 and T-cell activation (55). In addition, the biomarkers among OSCC clustered to more subgroups suggesting biological difference within OSCC; although we did not have significant differences between clustered groups in regards to demographics, tumor clinical-pathological characteristics, or outcome.

The limitations of the study should be considered. First, this is not a large-scale mass spectrometry or a microarray study to comprehensively interrogate the complex plasma proteome. Therefore, biological sources of variability in observed expression such as protein isoforms, or pre- or post-transcriptional modifications could not be identified. Instead, we wanted to apply a clinically translational platform to investigate the clinical value of immune-related biomarkers derived from easily accessible biosamples. Second, the retrospective nature of this study limits our full control over pre-analytical processing parameters, such as centrifugation time and speed, between laboratories, However, to our knowledge, there have been few reports of how centrifugation speed would significantly affect the detectable concentration of these proteins. A few studies may even suggest that plasma-derived proteins are relatively robust to various sample processing methods (56, 57). In regards to study population, the normal matched samples from the BCGP are the best available samples that are most representative of the general non-OSCC population with comprehensive data collections on demographics, smoking and no known any cancer history with follow-up. However, we do not have detailed medical information on whether there is presence of oral premalignant diseases, autoimmune diseases, and use of immunomodulators or other related conditions which could put this population at an increased risk of developing malignancies, which in turn may contribute to observed biomarker alterations. Third, 50% of the OSCC in this study was non-smokers, which is different from other geographic regions where tobacco-related OSCC remains high. Therefore, it is worth to note that our OSCC population may not be generalized. Lastly, the observed aberrantly expressed protein may be due to changes in metabolic states or other physiological states that could not be captured in this study. This limitation applies to all blood biomarker studies due to the varying genetic and non-genetic explanations, e.g., medical comorbidities and diets, in the population (58).

Future work is warranted to determine mechanisms by which most of these identified biomarkers are under-expressed in OSCC compared to normal. Other future directions may include a validation study with samples collected from different institutes with the determination of the best methods (e.g., ELISA vs. ECL) and cut-offs for various targets identified from this study. In addition, studies to investigate the temporal levels of these markers by repeating measurements before, post-treatment, and at time of local-regional recurrences or years into disease-free follow-up are of importance and can help to determine the value of these biomarkers in early identification of local and regional recurrence during the follow up.

In conclusion, this is the first paper to identify potential inflammatory plasma protein biomarkers of patients with OSCC. With further validation in larger sized cohort including paired blood samples collected over the course of disease management, the set of biomarkers has potential to assist in early detection of OSCC.

## Author Contributions

HK and CP contributed to the conception and design of the study. KL, XL, and YZ executed assay experiments and data acquisition. KL and NL performed the statistical analysis. KL wrote first draft of the manuscript. All authors interpreted and critically revised the manuscript to its final form. All authors gave final approval and agree to be accountable for all aspects of the work.

### Conflict of Interest Statement

The authors declare that the research was conducted in the absence of any commercial or financial relationships that could be construed as a potential conflict of interest.
