# Supplementary material for: Plasma-Derived Inflammatory Proteins Predict Oral Squamous Cell Carcinoma
Source: Front Oncol. 2018 Dec 4;8:585. doi: 10.3389/fonc.2018.00585 (PMC6288174; doi:10.3389/fonc.2018.00585)
Supplement: Supplementary file 1 [file Table_1.DOCX]

***Supplementary Material***

Plasma-derived inflammatory proteins predict oral squamous cell carcinoma

**Kelly Yi Ping Liu, Xian Jun David Lu, Yuqi Sarah Zhu, Nhu Le, Hugh Kim, Catherine Poh***

***Correspondence:** Corresponding Author: cpoh@dentistry.ubc.ca

1. **SUPPLEMENTARY TABLES**

| **Supplementary Table 1.** Patient demographic by study cohorts. | | | | | | | | |
| --- | --- | --- | --- | --- | --- | --- | --- | --- |
|  |  |  |  |  |  | **Cohort 2 comparison** | | |
| **Variables** | **Total samples (n=260)** | **Cohort 1 (OSCC, n=150)** | **Cohort 2 (OSCC, n=60; BCGP Normal n=50)** | ***p^b^*** |  | **BCGP Normal**  **(50)** | **OSCC**  **(60)** | ***p^b^*** |
| **Age, mean±SD** | 62.4±13.5 | 64.4±14.3 | 59.7±12.0 | 0.004 |  | 56.8±8.3 | 62.1±14.1 | 0.02 |
| **Age, median (1Q-3Qtile)** | 62 (53.8-71.4) | 65.2 (54.7-75.7) | 61 .0(50.2-66.0) |  |  | 60 (49-62) | 62.1 (53.1-72.5) |  |
|  |  |  |  |  |  |  |  |  |
| **Age Group** |  |  |  | 0.01 |  |  |  | 0.01 |
| <50 | 56 (21.5) | 28 (18.7) | 28 (25.5) |  |  | 14 (28) | 14 (23.3) |  |
| 50-62 | 77 (29.6) | 37 (24.7) | 40 (36.4) |  |  | 24 (48.0) | 16 (26.7) |  |
| >62 | 127 (48.8) | 85 (56.7) | 42 (38.2) |  |  | 12 (24.0) | 30 (50) |  |
|  |  |  |  |  |  |  |  |  |
| **Sex** |  |  |  | 1.0 |  |  |  | 0.45 |
| Male | 139 (53.5) | 80 (53.3) | 59 (53.6) |  |  | 29 (58.0) | 30 (50) |  |
| Female | 121 (46.5) | 70 (46.7) | 51 (46.4) |  |  | 21 (24.0) | 30 (50) |  |
|  |  |  |  |  |  |  |  |  |
| **Ethnicity** |  |  |  | 1.0 |  |  |  | 0.05 |
| White | 194 (74.6) | 112 (81.3) | 82 (74.5) |  |  | 42 (84.0) | 40 (66.7) |  |
| Others^a^ | 66 (25.4) | 38 (18.7) | 28 (25.5) |  |  | 8 (16.0) | 20 (33.3) |  |
|  |  |  |  |  |  |  |  |  |
| **Smoking History** |  |  |  | 0.45 |  |  |  | 0.73 |
| Never | 122 (46.9) | 66 (44.0) | 56 (50.9) |  |  | 25 (50.0) | 31 (51.7) |  |
| Current | 63 (24.2) | 37 (24.7) | 26 (23.6) |  |  | 14 (28.0) | 12 (20.0) |  |
| Former | 71 (27.3) | 45 (30.0) | 26 (23.6) |  |  | 11 (22.0) | 15 (25.0) |  |
| Unknown | 4 (5.2) | 2 (1.3) | 2 (1.8) |  |  |  | 2 (3.3) |  |

^a^Other ethnicity includes Aboriginal (n = 2) and Asian (n = 64)

^b^Statistical tests was performed excluding unknown data: Smoking History (n = 4).

Abbreviations: BCGP, British Columbia Generations Project; OSCC, oral squamous cell carcinoma

| **Supplementary Table 2.** Tumor characteristics among OSCC by study cohorts. | | | | |
| --- | --- | --- | --- | --- |
| **Variables** | **Total OSCC (n=210)** | **Cohort 1 OSCC (n=150)** | **Cohort 2 OSCC (n=60), excluding repeated samples** | ***p^b^*** |
| **Anatomical site** |  |  |  | 1.0 |
| Buccal Mucosa / hard palate | 17 (8.1) | 12 (8) | 5 (8.3) |  |
| Gingiva | 30 (14.3) | 20 (13.3) | 10 (16.7) |  |
| Soft Palate | 7 (3.3) | 4 (2.7) | 3 (5) |  |
| Floor of mouth | 16 (7.6) | 10 (6.7) | 6 (10) |  |
| Tongue | 140 (66.7) | 104 (69.3) | 36 (60) |  |
|  |  |  |  |  |
| **Clinical tumor size** |  |  |  | 0.78 |
| T1/T2 | 194 (92.3) | 139 (92.7) | 55 (91.7) |  |
| T3/T4 | 16 (7.6) | 11 (7.3) | 5 (8.3) |  |
|  |  |  |  |  |
| **Clinical nodal status** |  |  |  | 0.80 |
| cN0 | 185 (88.1) | 135 (90) | 53 (88.3) |  |
| cN+ | 22 (10.5) | 15 (10) | 7 (11.7) |  |
|  |  |  |  |  |
| **TNM Staging^a^** |  |  |  | 0.49 |
| Early-stage (T1/T2) | 152 (72.3) | 106 (70.7) | 46 (76.7) |  |
| Late-stage (T3/T4/N+) | 58 (27.6) | 44 (29.3) | 14 (23.3) |  |
|  |  |  |  |  |
| **Tumor grade** |  |  |  | 1.0 |
| Well | 54 (25.7) | 39 (26.0) | 15 (25.0) |  |
| Moderately | 118 (56.2) | 86 (57.3) | 32 (53.3) |  |
| Poorly | 34 (16.2) | 25 (16.7) | 9 (15.0) |  |
| Unknown | 4 (1.9) |  | 4 (6.7) |  |
|  |  |  |  |  |
| **DOI,** mean±SD; median (1Q-3Qtile) | 6.6±6.0;  5 (2-8.5) | 7.1±6.2;  5.5 (2.7-8.9) | 5.3±5.0;  4(1.6-8) | 0.04 |
| **DOI cut-off** |  |  |  | 0.02 |
| <4mm | 76 (36.2) | 47 (31.3) | 29 (48.3) |  |
| ≥4mm | 133 (63.3) | 103 (68.7) | 30 (50) |  |
| Unknown | 1 (0.5) |  | 1 (1.7) |  |
|  |  |  |  |  |
| **Concurrent END** |  |  |  | 0.24 |
| END-pN+ | 26 (12.4) | 19 (12.7) | 7 (11.7) |  |
| END-pN0 | 39 (18.6) | 32 (21.3) | 7 (11.7) |  |
| No END | 145 (69) | 99 (66) | 46 (76.7) |  |
|  |  |  |  |  |
| **LR** |  |  |  | 0.72 |
| 0 | 201 (95.7) | 144 (96) | 57 (95) |  |
| 1 | 9 (4.3) | 6 (4) | 3 (5) |  |
|  |  |  |  |  |
| **RF** |  |  |  | 0.18 |
| 0 | 149 (71.0) | 102 (68) | 47 (78.3) |  |
| 1 | 61 (29.0) | 48 (32) | 13 (21.7) |  |
|  |  |  |  |  |
| **Survival** |  |  |  | 0.004 |
| Alive | 149 (71.0) | 97 (64.7) | 52 (86.7) |  |
| Dead | 23 (10.9) | 21 (14) | 2 (3.3) |  |
| DOD | 38 (18.1) | 32 (21.3) | 6 (10) |  |
| ^a^OSCC patients were categorized based on the AJCC 8^th^ Edition Cancer Staging System for head and neck cancer implemented since January 2018. Early-stage OSCC consists of T1 or T2 with depth of invasion < 10mm; late-stage OSCC consists of T3 or any tumor >10 mm DOI, or T4, or lymph node positive.  ^b^statistical tests was performed excluding unknown data: Tumor Grade (n = 4); DOI (n = 1).  Abbreviations: OSCC, oral squamous cell carcinoma; cN0, clinical node-negative; clinical node-positive; DOI, depth of invasion; END, elective neck dissection; pN+, pathology node-positive; pN0, pathology node-negative; RF, regional failure to neck nodes, DOD, died of OSCC disease | | | | |

| **Supplementary Table 3.** Independent two-group Mann-Whitney test for screening 82 biomarkers in Cohort 1 and verification of 16 biomarkers in Cohort 2. | | | | | | |
| --- | --- | --- | --- | --- | --- | --- |
|  |  |  | **Cohort 1**  **(150 OSCC vs 10 normal)** | | **Cohort 2**  **(60 OSCC vs 50 BCGP normal)** | |
| **Biomarker** | **Common Names** | **Function** | ***p*** | ***p* (BH corrected)** | ***p*** | ***p* (BH corrected)** |
| **bFGF** | Basic fibroblast growth factor (FGF (basic)) | Growth factors | 5.8E-05 | **0.001** | 2.4E-08 | **3.7E-08** |
| **CRP** | C-reactive protein | Inflammatory marker | 4.0E-03 | **0.022** | 7.9E-02 | 7.9E-02 |
| CTACK | Cutaneous T-cell attracting chemokine / Chemokine (C-C motif) ligand 27 (CCL27) | Chemokine | 9.0E-03 | **0.046** |  |  |
| ENA78 | Epithelial-derived neutrophil-activating peptide / Chemokine (C-X-C) motif ligand 5 (CXCL5) | Chemokine | 7.0E-02 | 0.173 |  |  |
| Eotaxin | Chemokine (C-C motif) ligand 11 (CCL11) / Small-inducible cytokine A11 (SCYA11) | Chemokine | 3.5E-02 | 0.104 |  |  |
| Eotaxin2 | Chemokine (C-C motif) ligand 24 (CCL24) / Myeloid progenitor inhibitory factor 2 (MPIF-2) | Chemokine | 7.0E-01 | 0.851 |  |  |
| Eotaxin3 | Chemokine (C-C motif) ligand 26 (CCL26) / Macrophage inflammatory protein 4-alpha (MIP-4α) | Chemokine | 2.0E-01 | 0.367 |  |  |
| EPO | Erythropoietin | Cytokine | 9.0E-01 | 0.954 |  |  |
| Flt1 | FMS-like tyrosine kinase 1 / Vascular endothelial growth factor receptor 1 (VEGFR1) | Growth factors | 6.7E-01 | 0.835 |  |  |
| FLT3L | FMS-like tyrosine kinase 3 ligand | Cytokine, Growth factors | 7.9E-01 | 0.883 |  |  |
| Fractalkine | Chemokine (C-X3-C motif) ligand 1 (CX3CL1) | Chemokine | 8.3E-01 | 0.910 |  |  |
| GCSF | Granulocyte colony-stimulating factor | Cytokine | 1.0E+00 | 1.000 |  |  |
| GMCSF | Granulocyte-macrophage colony stimulating factor | Cytokine | 4.5E-01 | 0.657 |  |  |
| GROa | Human growth regulated alpha protein / CXCL1 | Chemokine | 7.2E-02 | 0.173 |  |  |
| **I309** | Chemokine (C-C motif) ligand 1 (CCL1) | Chemokine | 1.5E-05 | **0.001** | 1.0E-11 | **3.2E-11** |
| **ICAM1** | Intercellular adhesion molecule | Inflammatory marker | 7.1E-04 | **0.005** | 1.2E-09 | **2.8E-09** |
| IFNa2a | Interferon alpha 2 | Cytokine | 9.2E-01 | 0.966 |  |  |
| IFNb | Interferon beta | Cytokine | 9.5E-01 | 0.983 |  |  |
| IFNy | Interferon gamma | Cytokine | 1.1E-01 | 0.243 |  |  |
| **IL10** | Interleukin 10 | Cytokine | 2.9E-01 | 0.481 | 8.4E-09 | **1.7E-08** |
| IL12 | Interleukin-12p40/interleukin 23p40 | Cytokine | 7.8E-01 | 0.883 |  |  |
| IL12p70 | Interleukin 12p70 | Cytokine | 5.8E-02 | 0.158 |  |  |
| IL13 | Interleukin 13 | Cytokine | 6.5E-01 | 0.819 |  |  |
| IL15 | Interleukin 15 | Cytokine | 1.9E-01 | 0.367 |  |  |
| IL16 | Interleukin 16 / Lymphocyte chemoattractant factor (LCF) | Cytokine | 2.5E-01 | 0.445 |  |  |
| IL17A | Interleukin 17A / Cytotoxic T-lymphocyte-associated antigen 8 (CTLA-8) | Pro-inflammatory cytokine | 5.7E-01 | 0.758 |  |  |
| IL17AF | Interleukin 17A/interleukin 17F | Pro-inflammatory cytokine | 7.6E-01 | 0.877 |  |  |
| IL17B | Interleukin 17B | Pro-inflammatory cytokine | 1.7E-01 | 0.336 |  |  |
| IL17C | Interleukin 17C | Pro-inflammatory cytokine | 2.6E-01 | 0.451 |  |  |
| IL17D | Interleukin 17D | Pro-inflammatory cytokine | 1.0E-01 | 0.237 |  |  |
| IL17EIL25 | Interleukin 17E/interleukin 25 | Pro-inflammatory cytokine | 7.4E-01 | 0.870 |  |  |
| IL17F | Interleukin 17F | Pro-inflammatory cytokine | 1.5E-01 | 0.308 |  |  |
| IL18 | Interleukin 18 / Interferon-gamma inducing factor (IGIF) | Cytokine | 1.4E-02 | 0.055 |  |  |
| **IL1a** | Interleukin 1 alpha / Hematopoietin 1 | Cytokine | 3.0E-02 | 0.092 | 1.3E-13 | **1.0E-12** |
| IL1b | Interleukin 1 beta | Cytokine | 1.1E-01 | 0.245 |  |  |
| **IL1Ra** | Interleukin 1 receptor antagonist | Cytokine | 1.1E-04 | **0.001** | 1.9E-03 | **2.4E-03** |
| **IL2** | Interleukin 2 / T-cell growth factor (TCGF) | Cytokine | 3.0E-02 | 0.092 | 1.4E-12 | **7.4E-12** |
| IL21 | Interleukin 21 / Common variable immunodeficiency-11 (CVID11) | Cytokine | 3.1E-01 | 0.499 |  |  |
| IL22 | Interleukin 22 | Cytokine | 2.1E-01 | 0.387 |  |  |
| IL23 | Interleukin 23 | Cytokine | 3.3E-01 | 0.503 |  |  |
| IL27 | Interleukin 27 | Cytokine | 7.4E-01 | 0.870 |  |  |
| IL29 | Interleukin 29 / Interferon lambda 1 (IFNλ1) | Cytokine | 5.4E-01 | 0.753 |  |  |
| IL2Ra | Interleukin 2 receptor subunit alpha | Cytokine | 5.1E-01 | 0.733 |  |  |
| IL3 | Interleukin 3 | Cytokine | 7.2E-01 | 0.867 |  |  |
| IL31 | Interleukin 31 | Cytokine | 8.9E-01 | 0.954 |  |  |
| IL33 | Interleukin 33 | Cytokine | 3.9E-01 | 0.575 |  |  |
| IL4 | Interleukin 4 | Cytokine | 3.8E-01 | 0.574 |  |  |
| IL5 | Interleukin 5 / B-cell growth factor II (BCGF-II) / T-cell replacing factor (TRF) | Cytokine | 1.0E-02 | **0.047** |  |  |
| **IL6** | Interleukin 6 / B-cell stimulatory factor 2 (BSF-2) / CTL differentiation factor (CDF) / Hybridoma growth factor / Interferon beta-2 (IFN-β2) | Cytokine | 9.6E-03 | **0.046** | 2.6E-08 | **3.7E-08** |
| IL7 | Interleukin 7 | Growth factors | 2.0E-02 | 0.073 |  |  |
| IL8 | Interleukin 8 / Chemokine (C-X-C) motif ligand 8 (CXCL8) / Granulocyte chemotactic protein 1 (GCP-1) / Monocyte-derived neutrophil chemotactic factor (MDNCF) / Monocyte-derived neutrophil-activating peptide (MONAP) / Neutrophil-activating protein 1 (NAP-1) / Protein 3-10C / T-cell chemotactic factor | Chemokine | 3.2E-01 | 0.503 |  |  |
| IL9 | Interleukin 9 | Cytokine | 6.7E-02 | 0.173 |  |  |
| IP10 | Interferon gamma-induced protein 10 / Chemokine (C-X-C) motif ligand 10 (CXCL10) / Small-inducible cytokine B10 (SCYB10) | Chemokine | 1.2E-02 | 0.054 |  |  |
| ITAC | Interferon-stimulated T-cell alpha chemoattractant / Chemokine (C-X-C) motif ligand 11 (CXCL11) / Interferon gamma-inducible protein-9 (IP-9) | Chemokine | 5.7E-01 | 0.758 |  |  |
| MCP1 | Monocyte chemoattractant protein 1 / Chemokine (C-C motif) ligand 2 (CCL2) / Small inducible cytokine A2 (SCYA2) | Chemokine | 8.4E-04 | **0.005** |  |  |
| MCP2 | Monocyte chemoattractant protein 2 / Chemokine (C-C motif) ligand 8 (CCL8) | Chemokine | 1.7E-03 | **0.010** |  |  |
| **MCP3** | Monocyte-chemotactic protein 3 / Chemokine (C-C motif) ligand 7 (CCL7) | Inflammatory marker | 8.4E-06 | **0.001** | 5.9E-12 | **2.3E-11** |
| MCP4 | Monocyte-chemotactic protein 4 / Chemokine (C-C motif) ligand 13 (CCL13) / Small-inducible cytokine A13 (SCYA13) | Chemokine | 5.6E-04 | **0.005** |  |  |
| **MCSF** | Macrophage colony stimulating factor | Inflammatory marker | 8.3E-05 | **0.001** | 1.8E-08 | **3.2E-08** |
| MDC | Macrophage-derived chemokine / Chemokine (C-C motif) ligand 22 | Chemokine | 2.2E-02 | 0.076 |  |  |
| **MIF** | Macrophage migration inhibitory factor | Chemokine | 7.6E-05 | **0.001** | 4.2E-17 | **6.7E-16** |
| **MIP1a** | Macrophage inflammatory protein 1-alpha / Chemokine (C-C) motif ligand 3 (CCL3) | Chemokine | 9.7E-05 | **0.001** | 3.9E-11 | **1.0E-10** |
| MIP1b | Macrophage inflammatory protein 1-beta / Chemokine (C-C) motif ligand 4 (CCL4) | Chemokine | 2.9E-02 | 0.092 |  |  |
| MIP3a | Macrophage inflammatory protein 3-alpha / Chemokine (C-C) motif ligand 20 (CCL20) / Liver activation regulated chemokine (LARC) | Chemokine | 3.0E-01 | 0.484 |  |  |
| MIP3b | Macrophage inflammatory protein 3-beta / Chemokine (C-C) motif ligand 19 (CCL19) | Chemokine | 7.2E-02 | 0.173 |  |  |
| MIP5 | Macrophage inflammatory protein 5 / Chemokine (C-C) motif ligand 15 (CCL15) | Chemokine | 7.1E-04 | **0.005** |  |  |
| **SAA** | Serum amyloid A | Inflammatory marker | 5.6E-01 | 0.758 | 1.8E-03 | **2.4E-03** |
| SDF1a | Stromal cell-derived factor 1 / Chemokine (C-X-C) motif ligand 12 (CXCL12) | Chemokine | 4.6E-02 | 0.131 |  |  |
| TARC | Thymus and activation regulated chemokine / Chemokine (C-C) motif ligand 17 (CCL17) | Chemokine | 1.3E-02 | 0.055 |  |  |
| TGFB1 | Transforming growth factor beta 1 | Growth factors | 5.4E-01 | 0.753 |  |  |
| TGFB2 | Transforming growth factor beta 2 | Growth factors | 1.0E+00 | 1.000 |  |  |
| TGFB3 | Transforming growth factor beta 3 | Growth factors | 2.1E-02 | 0.073 |  |  |
| **Tie2** | Tyrosine kinase 2 | Growth factors | 5.1E-05 | **0.001** | 2.8E-03 | **3.2E-03** |
| TNFa | Tumor necrosis factor alpha / Cachectin | Cytokine | 6.1E-01 | 0.790 |  |  |
| TNFb | Tumor necrosis factor beta / Lymphotoxin-alpha (LT-alpha) | Cytokine | 2.2E-01 | 0.392 |  |  |
| TPO | Thrombopoietin / Megakaryocyte growth and development factor (MGDF) | Growth factors | 9.3E-02 | 0.217 |  |  |
| TRAIL | TNF-related apoptosis-inducing ligand | Cytokine | 9.6E-01 | 0.988 |  |  |
| TSLP | Thymic stromal lymphopoietin | Cytokine | 1.7E-01 | 0.336 |  |  |
| VEGFA | Vascular endothelial growth factor A | Growth factors | 1.9E-01 | 0.364 |  |  |
| VEGFC | Vascular endothelial growth factor C | Growth factors | 8.1E-01 | 0.895 |  |  |
| **VEGFD** | Vascular endothelial growth factor D | Growth factors | 3.8E-04 | **0.003** | 1.4E-02 | **1.5E-02** |
| YKL40 | Human cartilage glycoprotein 39 (HC-gp39) / Chitinase-3-like protein 1 (CHI3L1) | Inflammatory marker | 6.3E-01 | 0.806 |  |  |
| Bolded *p* values were significantly differentially expressed in OSCC after correcting for multiple testing with Benjamin-Hochberg (BH) method. Bolded biomarkers were selected verification in Cohort 2. | | | | | | |

| **Supplementary Table 4.** Patient demographics and clinical-pathological characteristics among OSCC (n = 210) by unsupervised clustering analysis. | | | | | |
| --- | --- | --- | --- | --- | --- |
| **Variables** | **Total OSCC (n=210)** | **Cluster Group 1 (n=24)** | **Cluster Group 2**  **(n=111)** | **Cluster Group 3**  **(n=75)** | ***p^c^*** |
| **Age, mean±SD** | 63.8±14.2 | 66.9±14.9 | 63.3±14.8 | 63.4±13.0 | 0.52 |
| **Age, median**  **(1Q-3Qtile)** | 63.7  (54.5-75.4) | 68.1  (56.9-79.6) | 62.8  (55.2-74.6) | 63.7  (54.5-740) |  |
|  |  |  |  |  |  |
| **Age Group** |  |  |  |  | 0.47 |
| <50 | 42 (20) | 5 (20.8) | 25 (22.5) | 12 (16) |  |
| 50-62 | 53 (25.2) | 4 (16.7) | 25 (22.5) | 24 (32) |  |
| >62 | 115 (54.8) | 15 (62.5) | 61 (55.0) | 39 (52) |  |
|  |  |  |  |  |  |
| **Sex** |  |  |  |  | 0.94 |
| Male | 110 (52.4) | 13 (54.2) | 57 (51.3) | 40 (53.3) |  |
| Female | 100 (47.6) | 11 (45.8) | 54 (48.7) | 35 (46.7) |  |
|  |  |  |  |  |  |
| **Ethnicity** |  |  |  |  | 0.39 |
| White | 152 (72.4) | 20 (83.3) | 77 (69.4) | 55 (73.3) |  |
| Others^a^ | 58 (27.6) | 4 (16.7) | 34 (30.6) | 20 (26.7) |  |
|  |  |  |  |  |  |
| **Smoking History** |  |  |  |  | 0.29 |
| Never | 49 (23.3) | 8 (33.3) | 59 (53.1) | 30 (40) |  |
| Current | 60 (28.6) | 6 (25) | 23 (20.7) | 20 (26.7) |  |
| Former | 97 (46.2) | 8 (33.3) | 27 (24.3) | 25 (33.3) |  |
| Unknown | 4 (1.9) | 2 (8.3) | 2 (1.8) |  |  |
|  |  |  |  |  |  |
| **Tumor site** |  |  |  |  | 0.42 |
| Buccal Mucosa / hard palate | 17 (8.1) | 2 (8.3) | 9 (8.1) | 6 (8) |  |
| Floor of mouth | 16 (7.6) | 2 (8.3) | 7 (6.3) | 7 (9.3) |  |
| Gingiva | 30 (14.3) | 3 (12.5) | 22 (19.8) | 5 (6.7) |  |
| Soft Palate | 7 (3.3) | 16 (66.7) | 4 (3.6) | 2 (2.7) |  |
| Tongue | 140 (66.7) |  | 69 (62.2) | 55 (73.3) |  |
|  |  |  |  |  |  |
| **Clinical tumor size** |  |  |  |  | 0.56 |
| T1/T2 | 194 (92.3) | 23 (95.8) | 100 (90.1) | 71 (95.7) |  |
| T3/T4 | 16 (7.6) | 1 (4.2) | 11 (9.9) | 4 (5.3) |  |
|  |  |  |  |  |  |
| **Clinical nodal status** |  |  |  |  |  |
| cN0 | 185 (88.1) | 22 (91.7) | 97 (87.4) | 69 (92) | 0.59 |
| cN+ | 22 (10.5) | 2 (8.3) | 14 (12.6) | 6 (8) |  |
|  |  |  |  |  |  |
| **TNM Staging^b^** |  |  |  |  | 0.005 |
| Early-stage (T1/T2) | 152 (72.4) | 21 (87.5) | 70 (63.1) | 61 (81.3) |  |
| Late-stage (T3/T4/N+) | 58 (28.6) | 3 (12.5) | 41 (36.9) | 14 (18.7) |  |
|  |  |  |  |  |  |
| **Tumor grade** |  |  |  |  | 0.64 |
| Well | 54 (25.7) | 6 (25) | 31 (28.0) | 17 (22.7) |  |
| Moderately | 118 (56.2) | 14 (58.3) | 62 (55.9) | 42 (56.0) |  |
| Poorly | 34 (16.2) | 2 (8.3) | 16 (14.4) | 16 (21.3) |  |
| Unknown | 4 (1.9) | 2 (8.3) | 2 (1.8) |  |  |
|  |  |  |  |  |  |
| **DOI,** mean±SD; median (1Q-3Qtile) | 6.6±6.0;  5 (2-8.5) | 5.1±3.7;  5 (1.8-8) | 6.8±5.8;  5 (2-10) | 6.7±6.7;  5 (2.9 – 8) | 0.46 |
|  |  |  |  |  |  |
| **DOI cut-off** |  |  |  |  |  |
| <4mm | 76 (36.2) | 10 (41.7) | 44 (39.6) | 22 (29.3) | 0.27 |
| ≥4mm | 133 (63.3) | 13 (54.2) | 67 (60.) | 53 (70.7) |  |
| Unknown | 1 (0.5) |  | 1 (0.9) |  |  |
|  |  |  |  |  |  |
| **Concurrent END** |  |  |  |  | 0.17 |
| END-pN+ | 26 (12.4) | 2 (8.33) | 18 (16.22) | 6 (8) |  |
| END-pN0 | 39 (18.6) | 3 (12.5) | 25 (22.52) | 11 (14.67) |  |
| No END | 145 (69) | 19 (79.17) | 68 (61.26) | 58 (77.33) |  |
|  |  |  |  |  |  |
| **LR** |  |  |  |  | 0.15 |
| 0 | 201 (95.7) | 21 (87.5) | 107 (96.4) | 73 (97.3) |  |
| 1 | 9 (4.3) | 3 (12.5) | 4 (3.6) | 2 (2.7) |  |
|  |  |  |  |  |  |
| **RF** |  |  |  |  | 0.35 |
| 0 | 149 (71.0) | 20 (83.3) | 78 (70.3) | 51 (68.0) |  |
| 1 | 61 (29.0) | 4 (16.7) | 33 (29.7) | 24 (32.0) |  |
|  |  |  |  |  |  |
| **Survival** |  |  |  |  | 0.47 |
| Alive | 149 (71.0) | 21 (87.5) | 78 (70.3) | 50 (66. 7) |  |
| Dead | 23 (10.9) | 1 (4.17) | 13 (11.7) | 9 (12.0) |  |
| DOD | 38 (18.1) | 2 (8.3) | 20 (18.0) | 16 (21.3) |  |
| Years to Alive, mean±SD | 3.66±1.89 | 2.86±2.07 | 3.38±1.83 | 4.43±1.66 | 0.05 |
| Years to Death, mean±SD | 2.63±1.58 | 5.49 | 2.59±1.47 | 2.37±1.59 | 0.2 |
| Years to DOD, mean±SD | 1.32±0.95 | 1.63±0.93 | 1.11±0.73 | 1.55±1.18 | 0.4 |
| ^a^Other ethnicity group include: Aboriginal (total, n=2; CG2, n=1; CG3, n=1), and Asian (total, n=56; CG1, n=4; CG2, n=33; CG3, n=19)  ^b^OSCC patients were categorized based on the AJCC 8^th^ Edition Cancer Staging System for head and neck cancers implemented since January 2018 (31). Early-stage OSCC consists of T1 or T2 with depth of invasion < 10mm; late-stage OSCC consists of T3 or any tumor >10 mm DOI, or T4, or lymph node positive.  ^c^Statistical tests was performed excluding unknown data: Tumor Grade (n = 4; CG1, n=2; CG2, n=2); DOI (n = 1, CG2, n=1).  Abbreviations: cN0, clinical node-negative; cN+, clinical node-positive; DOI, depth of invasion; END, elective neck dissection; pN+, pathology node-positive; pN0, pathology node-negative; LR, local recurrence; RF, regional failure to neck nodes; DOD, died of disease, OSCC. | | | | | |

| **Supplementary Table 5.** Differential expression analysis between early-stage OSCC and BCGP samples. | | | | | | | | | | |
| --- | --- | --- | --- | --- | --- | --- | --- | --- | --- | --- |
| Biomarker^a^ | Total (n=202) | |  | BCGP (n=50) | |  | Early-stage  OSCC (n=152)^b^ | |  | |
|  | Mean±SD | Median  (1Q-3Qtile) |  | Mean±SD | Median  (1Q-3Qtile) |  | Mean±SD | Median  (1Q-3Qtile) | Fold change^c^ | *p*^c^ |
| bFGF | 1.2±0.5 | 1.2  (0.9-1.6) |  | 1.6±0.3 | 1.6  (1.4-1.7) |  | 1.1±0.5 | 1.1  (0.8-1.4) | 0.62 | <0.0001 |
| CRP | 6.4±0.7 | 6.4  (6.0-6.9) |  | 6.7±0.6 | 6.7  (6.4-7.2) |  | 6.3±0.7 | 6.3  (5.9-6.7) | 0.75 | 0.06 |
| I309 | 1.7±0.2 | 1.7  (1.5-1.8) |  | 1.9±0.1 | 1.9  (1.8-2.0) |  | 1.6±0.2 | 1.6  (1.5-1.7) | 0.76 | <0.0001 |
| ICAM1 | 6.1±0.5 | 6.2  (5.8-6.5) |  | 6.2±0.3 | 6.3  (6.2-6.4) |  | 6.1±0.5 | 6  (5.8-6.5) | 0.67 | <0.0001 |
| IL10 | 0.3±0.2 | 0.2  (0.1-0.5) |  | 0.6±0.2 | 0.6  (0.5-0.7) |  | 0.2±0.2 | 0.1  (0.1-0.3) | 0.78 | <0.0001 |
| IL1a | 0.4±0.3 | 0.4  (0.2-0.6) |  | 0.6±0.2 | 0.7  (0.5-0.8) |  | 0.3±0.2 | 0.3  (0.2-0.4) | 0.64 | <0.0001 |
| IL1Ra | 2.2±0.2 | 2.2  (2.1-2.4) |  | 2.1±0.1 | 2.1  (2.1-2.2) |  | 2.3±0.2 | 2.3  (2.1-2.4) | 1.12 | 0.02 |
| IL2 | 0.4±0.4 | 0.2  (0.04-0.8) |  | 1.0±0.2 | 1.0  (08-1.1) |  | 0.2±0.3 | 0.1  (0-0.3) | 0.61 | <0.0001 |
| IL6 | 0.7±0.3 | 0.7  (0.6-0.9) |  | 1.0±0.2 | 1.0  (1.0-1.2) |  | 0.7±0.2 | 0.6  (0.5-0.8) | 0.75 | <0.0001 |
| MCP3 | 1.3±0.3 | 1.3  (1.1-1.5) |  | 1.5±0.2 | 1.6  (1.4-1.7) |  | 1.2±0.3 | 1.2  (1-1.4) | 0.63 | <0.0001 |
| MCSF | 1.2±0.2 | 1.1  (0.9-1.2) |  | 1.1±0.1 | 1.1  (1.0-1.2) |  | 1.1±0.2 | 1.1  (0.9-1.2) | 0.81 | <0.0001 |
| MIF | 4.4±0.2 | 4.5  (4.3-4.6) |  | 4.6±0.08 | 4.6  (4.6-4.7) |  | 4.3±0.2 | 4.3  (4.2-4.5) | 0.74 | <0.0001 |
| MIP1a | 1.9±0.4 | 2.0  (1.6-2.2) |  | 2.0±0.2 | 2.1  (1.9-2.2) |  | 1.9±0.4 | 1.8  (1.5-2.2) | 0.71 | <0.0001 |
| SAA | 6.7±0.7 | 6.7  (6.3-7.0) |  | 7.0±0.5 | 7.1  (6.8-7.3) |  | 6.5±0.7 | 6.5  (6.2-6.8) | 0.67 | <0.01 |
| Tie2 | 3.5±0.1 | 3.5  (3.4-3.6) |  | 3.6±0.1 | 3.6  (3.5-3.6) |  | 3.5±0.2 | 3.5  (3.4-3.6) | 0.94 | 0.04 |
| VEGFD | 2.8±0.2 | 2.8  (2.7-2.9) |  | 2.8±0.3 | 2.8  (2.7-2.9) |  | 2.8±0.2 | 2.8  (2.7-2.9) | 0.87 | <0.01 |
| ^a^Biomarker mean and median was calculated from log10 (pg/ml+1) transformed measurements.  ^b^ Early-stage OSCC consists of T1 or T2 with depth of invasion < 10mm based on the AJCC 8^th^ Edition Cancer Staging System for head and neck cancers implemented since January 2018 (31).  ^c^Differential expression analysis was adjusted for age group, ethnicity, and batch-effect. | | | | | | | | | | |

| **Response Table 3**. Biomarker concentration between early-stage and late-stage OSCC | | | | | | |
| --- | --- | --- | --- | --- | --- | --- |
|  | **Early-stage OSCC (n=152)^b^** | |  | **Late-stage OSCC (n=58)^b^** | |  |
| **Biomarker^a^** | **Mean±SD** | **Median(Q1-Q3)** |  | **Mean±SD** | **Median(Q1-Q3)** | ***p*** |
| bFGF | 1.1±0.5 | 1.1 (0.8-1.4) |  | 1.0±0.6 | 0.9 (0.6-1.2) | 0.02 |
| CRP | 6.3±0.7 | 6.3 (5.9-6.7) |  | 6.7±0.7 | 6.7 (6.2-7.1) | <0.001 |
| I309 | 1.6±0.2 | 1.6 (1.5-1.7) |  | 1.6±0.2 | 1.6 (1.4-1.7) | 0.11 |
| ICAM1 | 6.1±0.5 | 6 (5.8-6.5) |  | 6±0.4 | 5.9 (5.8-6.2) | 0.14 |
| IL10 | 0.2±0.2 | 0.1 (0.1-0.3) |  | 0.2±0.2 | 0.2 (0.1-0.3) | 0.18 |
| IL1a | 0.3±0.2 | 0.3 (0.2-0.4) |  | 0.4±0.2 | 0.3 (0.2-0.5) | 0.86 |
| IL1Ra | 2.3±0.2 | 2.3 (2.1-2.4) |  | 2.4±0.3 | 2.3 (2.2-2.5) | 0.04 |
| IL2 | 0.2±0.3 | 0.1 (0-0.3) |  | 0.2±0.2 | 0.1 (0.1-0.2) | 0.39 |
| IL6 | 0.7±0.2 | 0.6 (0.5-0.8) |  | 0.7±0.3 | 0.7 (0.5-0.8) | 0.15 |
| MCP3 | 1.2±0.3 | 1.2 (1-1.4) |  | 1.1±0.2 | 1.1 (1-1.3) | 0.06 |
| MCSF | 1.1±0.2 | 1.1 (0.9-1.2) |  | 1.1±0.2 | 1.1 (0.9-1.2) | 0.47 |
| MIF | 4.3±0.2 | 4.3 (4.2-4.5) |  | 4.3±0.3 | 4.3 (4.1-4.5) | 0.50 |
| MIP1a | 1.9±0.4 | 1.8 (1.5-2.2) |  | 1.7±0.4 | 1.6 (1.5-2) | 0.03 |
| SAA | 6.5±0.7 | 6.5 (6.2-6.8) |  | 6.9±0.7 | 6.9 (6.4-7.5) | <0.01 |
| Tie2 | 3.5±0.2 | 3.5 (3.4-3.6) |  | 3.4±0.2 | 3.5 (3.4-3.5) | 0.10 |
| VEGFD | 2.8±0.2 | 2.8 (2.7-2.9) |  | 2.7±0.2 | 2.8 (2.6-2.9) | 0.25 |
| ^a^Biomarker mean and median was calculated from log10 (pg/ml+1) transformed measurements.  ^b^OSCC patients were categorized based on the AJCC 8^th^ Edition Cancer Staging System for head and neck cancers implemented since January 2018 (31). Early-stage OSCC consists of T1 or T2 with depth of invasion < 10mm; late-stage OSCC consists of T3 or any tumor >10 mm DOI, or T4, or lymph node positive. | | | | | | |
